# Weather impacts on interactions between nesting birds, nest-dwelling ectoparasites and ants

**DOI:** 10.1038/s41598-022-21618-1

**Published:** 2022-10-25

**Authors:** Marta Maziarz, Richard K. Broughton, Przemysław Chylarecki, Grzegorz Hebda

**Affiliations:** 1 Museum and Institute of Zoology, Polish Academy of Sciences, Wilcza 64, 00-679 Warsaw, Poland; 2 UK Centre for Ecology & Hydrology, Maclean Building, Benson Lane, Crowmarsh Gifford, Wallingford, OX10 8BB UK; 3 Institute of Biology, University of Opole, Oleska 22, 45-052 Opole, Poland

**Keywords:** Zoology, Ecology, Community ecology

## Abstract

Weather has a dominant impact on organisms, including their life histories and interspecific interactions. Yet, for nesting birds, and the arthropods inhabiting bird nests, the direct and cascading effects of weather are poorly known. We explored the influence of ambient temperatures and rainfall on the cohabitation of dome-shaped bird nests by Wood Warblers *Phylloscopus sibilatrix*, their blowfly *Protocalliphora azurea* ectoparasites, and predatory *Myrmica* and *Lasius* ants that may provide nest sanitation. We sampled blowflies and ants in 129 nests, and measured warbler nestlings during 2018–2020 in the primeval Białowieża Forest, eastern Poland. The probability of ectoparasites occurring in nests increased with increasing ambient temperatures and declining precipitation in the early nestling stage, when adult blowflies are ovipositing. Where present, the number of ectoparasites was greater if higher ambient temperatures had prevailed in the late nestling stage, but only when ants were absent from nests. However, the nestling growth was unrelated to ectoparasite abundance or ant presence within bird nests, although it was lower at high rainfall. The results suggest that weather can have conflicting impacts on interactions between nesting birds and nest-dwelling arthropods, but birds can mostly compensate for any related costs in old-growth forest, where food is generally abundant.

## Introduction

Climate and weather have intricate effects on organisms, with direct implications for their life histories, abundance and distributions, but also reciprocal indirect effects via interspecific interactions^1–3^. As individual species vary in their requirements and tolerance to weather, any variation in ambient conditions may cause divergent responses that affect interspecific interactions^4,5^. Due to the complexity of interaction networks between organisms^6^, predicting the directions and outcomes of changes in weather can be challenging, yet is crucial in the face of climate change and shifting weather patterns^3^.

Weather is known to have potential cascading effects via ecological links between species within broad habitats and, in extreme situations, to cause significant shifts of ecosystem functioning. Such examples from different types of ecological networks include food webs, mutualistic relationships and host-parasite networks^2,3,7,8^. However, similar analyses are scarce for microenvironments, such as bird or mammal nests, which may host a diversity of interconnected species, and function as ‘microecosystems’.

The nests of endothermic birds and mammals are inhabited by a rich fauna of arthropods, comprising predators, scavengers and ectoparasites^9,10^. These communities may form complex ecological networks within nests, and also interact with the hosts^9,11,12^. Although bird and mammal nests are composed of insulative materials to buffer the owners against ambient conditions^12,13^, weather might still influence the hosts and the arthropod community within the nests. Therefore, as with impacts on species in broad habitats, the cascading effects of weather (e.g. ambient temperatures or rainfall) may also affect coexisting species on this smaller scale within the microenvironment of a nest.

The arthropods inhabiting bird nests are relatively well studied, and most research has focused on relationships between nesting birds and nest-dwelling ectoparasites, such as blowflies *Protocalliphora* spp. (Diptera: Calliphoridae) (89 papers published since 1977 according to Web of Science accessed on 23 February 2022; key words: (“bird” OR “nest”) AND “blowfly”). Blowfly larvae or pupae have been recorded in the nests of hundreds of bird species across the Holarctic^14^, but systematic studies of blowfly infestation come almost exclusively from cup-shaped nests built inside cavities, mainly nest-boxes. Similar research is generally lacking for songbirds that build dome-shaped nests which are unenclosed within a tree or rock cavity, and so are directly exposed to ambient conditions.

The few studies comparing nest microclimates indicate that the insulation of dome-shaped nests is similar to unenclosed, open-cup nests^15–17^, contradicting assumptions of a more stable microclimate in the former^18–20^. For example, measurements of the dome-shaped nests of Wood Warblers *Phylloscopus sibilatrix* showed large daily temperature fluctuations within the nest walls (mean daily internal amplitudes ranging between 4 and 13 °C, at mean daily ambient amplitudes of 6–16 °C), with internal temperatures that strongly depended on ambient conditions^21^. Thus, the hosts and the arthropods inhabiting unenclosed, dome-shaped nests could be greatly exposed to variable weather, despite some protection provided by the roof against direct solar radiation or rainfall. Nevertheless, more studies on the microclimate of dome-shaped nests are necessary to assess their thermal and humidity properties in comparison to unenclosed, open-cup nests.

It has been suggested that nest infestation by blowflies could be hindered by cool and wet weather^22–24^. Low ambient temperatures and heavy rainfall might reduce the activity of adult blowflies ovipositing in bird nests^25–27^, presumably resulting in fewer nests being parasitised. Despite the thermal activity of birds within their nests^21,28^, cool weather might also negatively affect the nest microclimate for developing blowfly larvae. The period of blowfly larvae development may be shortest at nest temperatures of *c*. 35 °C^27^, while lower nest temperatures (e.g. 13–18 °C) would extend the larval stage^27^, and potentially reduce ectoparasite numbers in cooler nests^29^. As such, cool and wet weather might limit blowfly nest infestation and the negative impact of ectoparasites on hosts^29,30^. Other reasons for varying nest infestation by blowflies may include the brood size of hosts, variation between habitats, and/or other undefined factors related to annual and within-season variation^24,26,29,31–33^. Nest sanitation by birds actively searching for ectoparasites in the nesting material may also be important^34^.

As bird nests are inhabited by communities of nest-dwelling arthropods^9^, weather might also influence ectoparasite infestation indirectly, via potential predator–prey interactions. For example, ants can act as predators and reduce the number of nest-dwelling ectoparasites, such as bugs *Oeciacus vicarius* (Heteroptera: Cimicidae) or flies *Carnus hemapterus* (Diptera: Carnidae)^35,36^. As the foraging activity of ant workers can be promoted by warm weather^37,38^, the sanitation of bird nests by ants might be more effective at higher ambient temperatures, potentially having subsequent fitness consequences for the host birds.

Warm and dry weather may also have a positive influence on the host birds by, for example, increasing the availability of food and promoting the growth or survival of nestlings^39–41^. However, this benefit might be limited by the increased ectoparasite (blowfly) infestation of bird nests during warm and dry weather, although this, in turn, might then be mitigated by increased foraging by predatory ants (see above). Other factors, such as habitat type, brood size or nestling age, might moderate the influence on birds of the ambient conditions, ectoparasites or ants^40,42–45^. Therefore, a holistic approach is needed to unravel the intricacy of host responses and interspecific interactions to convoluted weather effects within the microenvironments of bird nests.

In this study, we investigated the influence of weather on interactions between nesting Wood Warblers (songbird hosts), blowflies *Protocalliphora azurea* (Fallén, 1817; ectoparasites), and *Lasius* or *Myrmica* ants (predators of arthropods), which all cohabit in the birds’ dome-shaped nests. First, we assessed the occurrence and intensity of blowfly infestation in the bird nests. We then tested whether the ectoparasite infestation varied with changing ambient temperatures and rainfall in the early nestling period, when chicks were young and female adult blowflies would be ovipositing in the bird nests^27^. We repeated these tests for the late nestling period, when chicks were older and the blowfly larvae would be developing within bird nests^14^. Additionally, we checked whether reduced occurrence or intensity of ectoparasite infestation was associated with the presence of ants at a range of ambient conditions, when the foraging activity of ant workers may vary^37,38^. We also explored the fitness consequences for birds, expressed as the variation in the growth (feather length and body mass) in relation to weather, blowfly parasitism, or ant presence within the nests. Co-variables of habitat type, study year, bird nest phenology, brood size and nestling age were considered in explaining the degree of blowfly infestation or nestling growth, hypothetically moderating the observed patterns. Finally, we compared the mortality of nestlings (brood reduction between nestlings hatching and leaving the nests) in the nests with and without blowflies.

## Results

### The occurrence and intensity of ectoparasite infestation

Blowflies were the near-exclusive nest-dwelling ectoparasites in the Wood Warbler nests in the Białowieża National Park, eastern Poland. Only one nest contained a single flea (Siphonaptera), and in ten nests we found single ticks (Ixodidae).

The occurrence of blowflies was generally low, being recorded in 17 of 49 nests in 2018, seven of 26 nests in 2019, and six of 54 nests in 2020. The number of blowflies per infested nest averaged 29.6 (95% CIs: 21.3–38.1, range: 1–63, *n* = 17 nests) in 2018, 32.0 (95% CIs: 11.7–57.9, range: 3–97, *n* = 7) in 2019 and 33.7 (95% CIs: 26.8–44.0, range: 24–58, *n* = 6) in 2020.

**Figure 1:**
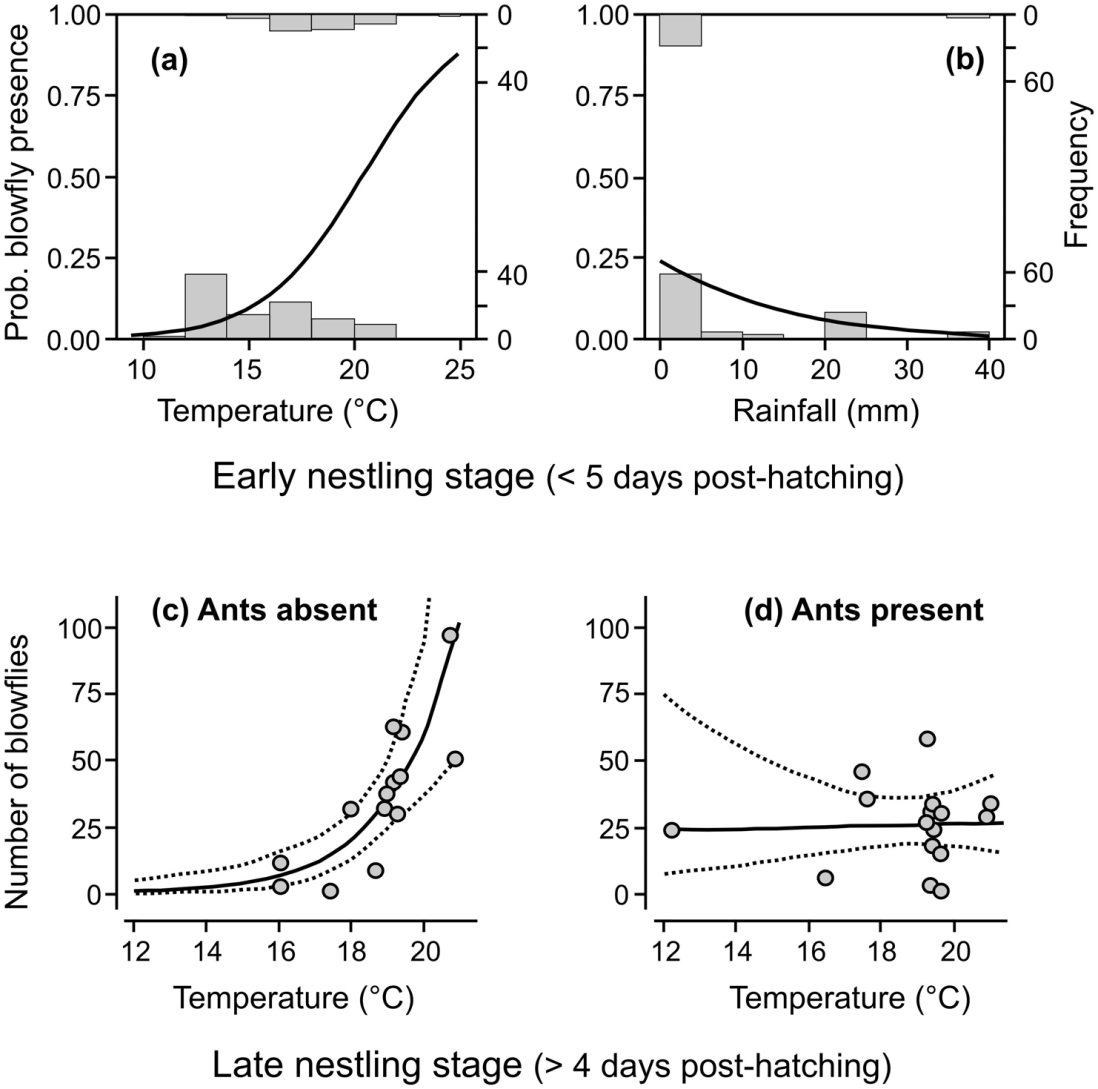
The likelihood of blowfly *Protocalliphora azurea* presence in relation to weather, (**a**) mean ambient temperature and (**b**) total sum of rainfall, in the early nestling stage of Wood Warblers *Phylloscopus sibilatrix* (*n* = 129 nests). Additionally, changes in blowfly number in parasitised nests with increasing mean ambient temperature in the late nestling stage: (**c**) nests without ants (*n* = 14 nests) and (**d**) nests with ants (*n* = 16 nests). Solid lines represent trends, and dashed lines are 95% confidence intervals (CIs) of hurdle models with ΔAICc = 0.00 (Table 1; package pscl^92,93^). Shown are histograms of the number (frequency) of nests containing blowflies (upper) and those without blowflies (bottom) (grey bars in panel **a** and **b**).

The occurrence and intensity of blowfly infestation varied with weather, but the relationship differed depending on ambient conditions in the early or late nestling stage. In the early nestling stage, when nestlings were 1–4 days old, the likelihood of blowfly presence increased with the mean ambient temperature and decreasing sum of rainfall (Fig. 1a,b; Tables 1, 2). However, in the late nestling stage, the relationship between the occurrence of blowflies and weather conditions was weak; only the sum of rainfall was retained in the binomial part of hurdle models with ΔAICc < 2 (Table 1), but became insignificant in averaged models (Table 2).

**Table 1:** Selection of hurdle negative binomial models (ΔAICc < 2) testing blowfly *Protocalliphora azurea* infestation of 129 Wood Warbler *Phylloscopus sibilatrix* nests (response variable) depending on weather in early or late nestling stages.

Model (count | binomial part)	df	Log(L)^h^	AICc	ΔAICc	Weights
**Weather in early nestling stage (nestlings < 5 days old)**					
Hatching date^a^ | temperature^b^ + rain^c^	6	− 183.7	380.1	0.00	0.015
Hatching date | temperature + rain + brood size ^d^	7	− 182.9	380.7	0.61	0.011
Habitat type^e^ | temperature + rain	6	− 184.4	381.5	1.36	0.008
Hatching date | temperature + rain + ants^f^	7	− 183.3	381.5	1.42	0.008
Hatching date + habitat | temperature + rain	7	− 183.4	381.6	1.52	0.007
Hatching date | temperature + rain + habitat	7	− 183.4	381.7	1.58	0.007
Hatching date | temperature + rain + ants + brood size	8	− 182.3	381.8	1.73	0.007
Hatching date + ants | temperature + rain	7	− 183.5	382.0	1.92	0.006
Habitat | temperature + rain + brood size	7	− 183.6	382.1	1.97	0.006
**Weather in late nestling stage (nestlings > 4 days old)**					
Temperature + ants + temp. × ants | year ^g^ + hatching date + rain	10	− 181.4	384.6	0.00	0.080
Temperature + ants + temp. × ants | year + hatching date	9	− 183.4	386.3	1.67	0.035
Temperature + ants + temp. × ants + brood size | year + hatching date	10	− 182.3	386.4	1.83	0.032

The count part with negative binomial distribution estimated changes in the number of blowflies in infested nests, and the hurdle binomial tested the likelihood of blowfly presence (package pscl^92,93^). A null model had only fixed intercepts on the count and binomial parts.

^a^Relative hatching date of nestlings (days from the median hatching date in a year); ^b^an average of daily means (°C); ^c^a sum of daily sums (mm); ^d^the number of nestlings hatched; ^e^habitat type (deciduous or mixed deciduous-coniferous); ^f^presence or absence of ants in bird nests (as a factor); ^g^study year (2018–2020; as a factor); ^h^log-likelihood.

**Table 2:** Model-averaged covariates best explaining the number of blowflies in infested nests of Wood Warblers (count), and the likelihood of blowfly presence (binomial) in nests (package pscl^92,93^).

Variable	Weather in early nestling stage				Weather in late nestling stage			
	estimate	s.e.m	z	95% CI	estimate	s.e.m	z	95% CI
**Count part with negative binomial distribution**								
Intercept	**3.34**	**0.34**	**9.7**	**2.67, 4.02**	− **6.64**	**2.82**	**2.4**	− **12.16, **− **1.11**
Temperature	NA	NA	NA	NA	**0.56**	**0.14**	**3.9**	**0.28, 0.84**
Ants (present)	− 0.17	0.29	0.6	− 0.74, 0.41	**10.21**	**3.14**	**3.2**	**4.05, 16.38**
Temp. × ants (present)	NA	NA	NA	NA	− **0.55**	**0.17**	**3.3**	− **0.88, **− **0.23**
Hatching date	**0.05**	**0.02**	**2.0**	**0.00, 0.09**	NA	NA	NA	NA
Habitat (deciduous)	− 0.50	0.38	1.3	− 1.25, 0.24	NA	NA	NA	NA
Brood size	NA	NA	NA	NA	− 0.32	0.22	1.5	− 0.74, 0.11
Log(theta)	0.57	0.30	1.9	− 0.02, 1.17	**0.93**	**0.31**	**3.0**	**0.31, 1.55**
**Binomial (hurdle) part**								
Intercept	− **8.91**	**2.87**	**3.1**	− **14.55, **− **3.28**	− **0.87**	**0.33**	**2.6**	− **1.52, **− **0.22**
Temperature	**0.43**	**0.12**	**3.7**	**0.20, 0.65**	NA	NA	NA	NA
Rain	**− 0.08**	**0.03**	**2.4**	− **0.14, **− **0.01**	0.06	0.03	1.9	0.00, 0.12
Year (2019)	NA	NA	NA	NA	− 0.67	0.60	1.1	− 1.84, 0.50
Year (2020)	NA	NA	NA	NA	− **2.57**	**1.08**	**2.4**	− **4.69, **− **0.45**
Hatching date	NA	NA	NA	NA	**0.19**	**0.06**	**3.2**	**0.08, 0.30**
Ants (present)	0.48	0.49	1.0	− 0.49, 1.44	NA	NA	NA	NA
Habitat (deciduous)	− 0.55	0.67	0.8	− 1.87, 0.77	NA	NA	NA	NA
Brood size	0.43	0.34	1.3	− 0.23, 1.10	NA	NA	NA	NA

Shown are (conditional) model-averaged coefficients and confidence intervals (CI) across top models (ΔAICc < 2) with different sets of covariates on count and binomial parts (Table 1). Significant relationships (*P* < 0.05) are marked in bold; NAs—covariates dropped during model selection (present in models with ΔAICc ≥ 2).

When mean ambient temperature in the late nestling stage was dropped from the hurdle binomial models with ΔAICc < 2 (Table 1), the likelihood of blowfly presence appeared lowest in 2020, and generally increased in broods initiated later in a season (Table 2). The likelihood of blowfly presence was weakly related to other factors, such as ant presence (lone foraging workers and any associated larvae or pupae), Wood Warbler brood size, or habitat type; the variables were excluded from the top models (ΔAICc < 2) or became insignificant in the binomial part of averaged models (Tables 1, 2).

The number of blowflies in infested nests seemed unrelated to weather conditions in the early nestling stage, as both weather variables were excluded from the count part of models with ΔAICc < 2 (Table 1). Notably, blowflies were more numerous in parasitised nests when exposed to higher mean ambient temperatures in the late nestling stage, but only if ants were absent (Fig. 1c). When ants were present in the same nests, the intensity of blowfly infestation remained mostly unchanged at the range of ambient temperatures (Fig. 1d). The two-way interaction term between ant presence and ambient temperature in the late nestling stage was significant in the count part of averaged models with ΔAICc < 2 (Tables 1, 2). The total sum of rainfall in the late nestling stage was dropped from the count part of models with ΔAICc < 2, indicating a weak relationship with the number of blowflies in parasitised nests (Table 1). Overall, the intensity of blowfly infestation tended to be lower in nests containing ants, averaging 26.0 blowfly larvae or pupae (95% CIs: 18.8–33.2, *n* = 16 nests), compared to a mean of 36.8 (95% CIs: 24.0–50.6, *n* = 14) in nests without ants.

The number of blowflies in infested nests tended to be higher in broods initiated later in a season (Tables 1, 2). However, the relationship became significant only when the mean ambient temperature in the early nestling stage was dropped from the count part of models with ΔAICc < 2 (Tables 1, 2). There was little variation in the intensity of blowfly infestation in relation to study year, habitat type, or brood size, as these variables were dropped from the count part of models with ΔAICc < 2, or became insignificant if retained (Tables 1, 2).

### The growth of Wood Warbler nestlings

**Figure 2:**
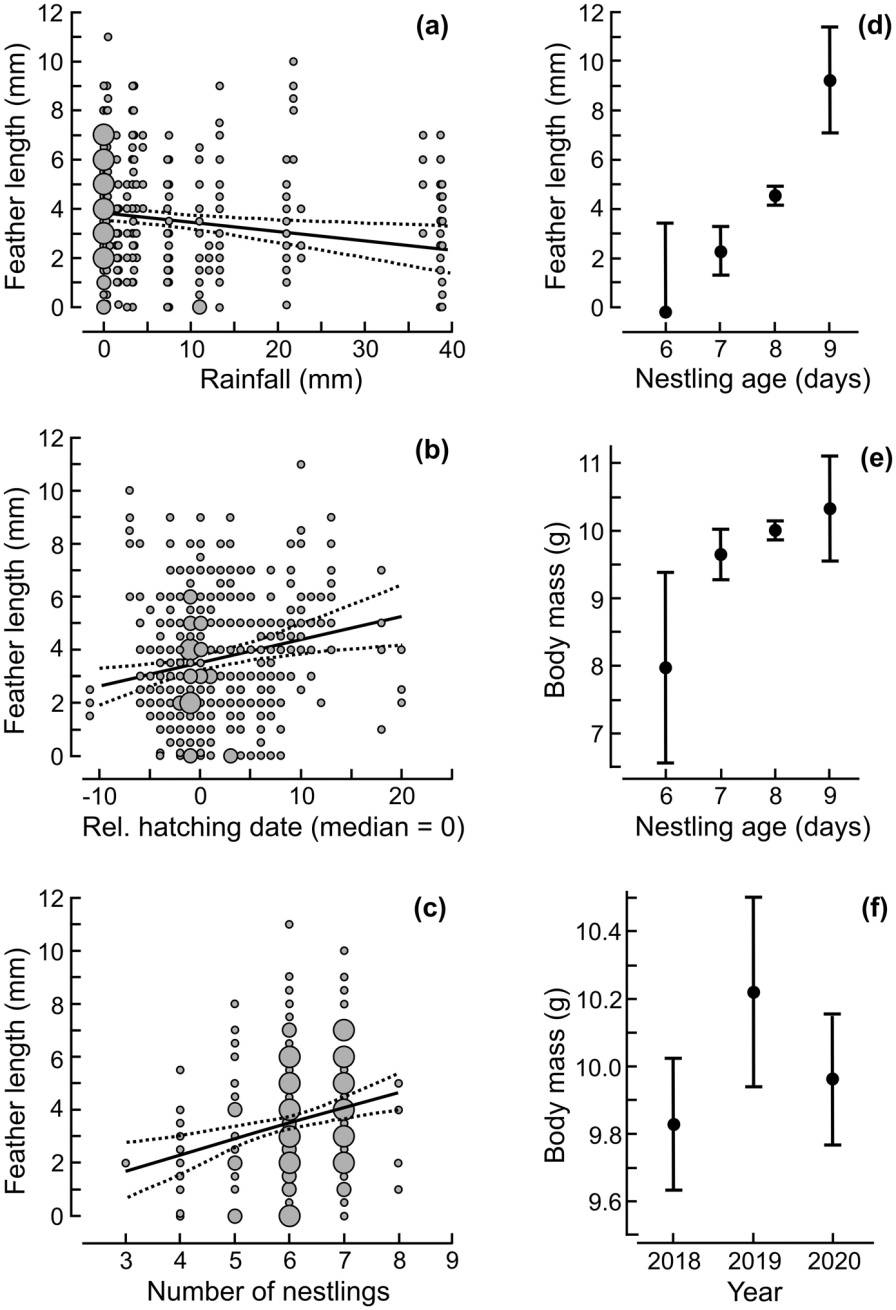
The length of the 3rd primary feather or body mass of Wood Warbler nestlings in relation to (**a**) the total sum of rainfall in the period when nestlings were > 4 days old until their measurement at median 8 days old; (**b**) relative hatching date (days from median hatching date in a year), (**c**) brood size (number of nestlings per nest), (**d**–**e**) nestling age since hatching, and (**f**) year. Solid lines indicate trends (**a**–**c**), black dots are means (**d**–**f**), and dashed lines or whiskers are the 95% CIs from the models with ΔAICc = 0.00 (Tables 3 and 5). The size of grey dots indicates sample sizes: small (1–10 nestlings measured), medium (11–20), large (> 20 nestlings). In total, 742 nestlings were measured from 129 nests in 2018–2020.

The length of nestling primary feathers tended to be shorter with an increasing total amount of rainfall during the period from when the nestlings were 5 days old and until they were measured at a median 8 days old (Fig. 2a; Tables 3, 4). There was an insignificant relationship between feather length and mean ambient temperature in the same period, as the temperature was dropped from the top linear mixed effects models with ΔAICc < 2 (Table 3). The body mass of nestlings changed little in relation to ambient temperatures and rainfall during the period from when they were 5 days old and until they were measured (both weather variables were insignificant in averaged models; Tables 5, 6).

**Table 3:** Selection of linear mixed effect models (ΔAICc < 2) best explaining variation in the length of the 3rd primary feather (mm) of Wood Warbler nestlings (response variable; *n* = 129 nests).

Models	df	Log(L)^f^	AICc	ΔAICc	Weights
Rain^a^ + hatching date^b^ + brood size^c^ + nestling age^d^	9	− 1389.7	2797.5	0.00	0.204
Blowfly abundance^e^ + rain + hatching date + brood size + nestling age	10	− 1389.6	2799.5	1.91	0.079

The remaining covariates also included in the global model, but dropped during model selection, were: the average mean daily ambient temperature (°C) in the period when nestlings were > 4 days old until their measurement (at median 8 days old), ant presence (as a factor), habitat type (deciduous or mixed deciduous-coniferous) and study year (as a factor). A null model had only a fixed intercept, and a random effect was the individual nest.

^a^The total sum of rainfall (mm) in the nestling stage when nestlings were > 4 days old until their measurement; ^b^relative hatching date of Wood Warbler nestlings (days from the median hatching date in a year); ^c^the number of nestlings hatched; ^d^the number of days since hatching (day 0; set as a factor); ^e^the number of blowfly larvae or pupae per nestling in nests with and without blowflies; ^f^log- likelihood.

**Table 4:** Model-averaged variables best explaining the length of the 3rd primary feather (mm) of Wood Warbler nestlings (response variable).

Variables	Estimate	s.e.m	z	CI 2.5%	CI 97.5%
**Intercept**	− **3.81**	**1.86**	**2.0**	**− 7.461**	**− 0.160**
Blowfly abundance	0.02	0.06	0.4	− 0.093	0.137
**Rain**	**− 0.04**	**0.01**	**2.7**	**− 0.064**	**− 0.010**
**Hatching date**	**0.09**	**0.03**	**2.8**	**0.027**	**0.148**
**Brood size**	**0.59**	**0.17**	**3.5**	**0.262**	**0.920**
**Nestling age**					
7 days	2.16	1.61	1.3	− 1.012	5.331
**8 days**	**4.04**	**1.57**	**2.6**	**0.951**	**7.131**
**9 days**	**7.94**	**1.83**	**4.3**	**4.356**	**11.529**

Shown are averaged coefficients and confidence intervals (CI) across models with ΔAICc < 2 (Table 3; conditional average). Significant relationships (*P* < 0.05) are marked in bold. For the description of variables see Table 3.

**Table 5:** Selection of linear mixed effect models (ΔAICc < 2) best explaining the variation in body mass (g) of 742 Wood Warbler nestlings from 129 nests (response variable).

Models	df	Log(L)	AICc	ΔAICc	weights
Year + nestling age	8	− 907.3	1830.8	0.00	0.034
Temperature + nestling age	7	− 908.3	1830.8	0.06	0.033
Temperature + year + nestling age	9	− 906.5	1831.2	0.38	0.028
Nestling age	6	− 909.8	1831.7	0.90	0.022
Year + hatching date + nestling age	9	− 906.9	1832.0	1.27	0.018
Year + hatching date + brood size	9	− 907.1	1832.4	1.63	0.015
Rain + year + nestling age	9	− 907.1	1832.4	1.64	0.015
Temperature + brood size + nestling age	8	− 908.1	1832.4	1.65	0.015
Temperature + rain + nestling age	8	− 908.3	1832.7	1.92	0.013
Year + nestling age + habitat type	9	− 907.2	1832.7	1.96	0.013
Blowfly abundance + year + nestling age	9	− 907.3	1832.7	1.97	0.013
Blowfly abundance + temperature + nestling age	8	− 908.3	1832.8	1.99	0.013
Temperature + hatching date + nestling age	8	− 908.3	1832.8	2.00	0.013

The remaining covariate also included in the global model, but dropped during model selection, was ant presence (as a factor). A null model had only a fixed intercept, and a random effect was the nest identity. For the description of covariates see Table 3.

**Table 6:** Model-averaged variables best explaining the body mass (g) of Wood Warbler nestlings (response variable).

Variables	Estimate	s.e.m	z	CI 2.5%	CI 97.5%
**Intercept**	**8.21**	**0.87**	**9.4**	**6.499**	**9.925**
Blowfly abundance	0.00	0.03	0.0	− 0.049	0.050
Temperature	− 0.03	0.02	1.6	− 0.070	0.008
Rain	0.00	0.01	0.5	− 0.018	0.010
**Year (2019)**	**0.38**	**0.18**	**2.1**	**0.030**	**0.731**
Year (2020)	0.11	0.16	0.7	− 0.196	0.425
Hatching date	0.00	0.02	0.3	− 0.038	0.029
Brood size	− 0.05	0.08	0.7	− 0.203	0.100
**Nestling age**					
**7 days**	**1.70**	**0.74**	**2.3**	**0.241**	**3.161**
**8 days**	**2.01**	**0.72**	**2.8**	**0.590**	**3.425**
**9 days**	**2.28**	**0.83**	**2.7**	**0.652**	**3.914**
Habitat type (deciduous)	0.06	0.19	0.3	− 0.307	0.421

Shown are averaged coefficients and confidence intervals (CI) across models with ΔAICc < 2 (Table 5; conditional average). Significant relationships (*P* < 0.05) are marked in bold. For the description of covariates see Table 3.

The growth of Wood Warbler nestlings was weakly related to blowfly abundance, i.e. the number of ectoparasites per nestling in all 129 nests with and without blowflies. Although some of the models that best explained the change in length of primary feathers or body mass of nestlings did include blowfly abundance (Tables 3 and 5), this variable was insignificant in averaged models (Tables 4 and 6). The nestling growth (feather length and body mass) appeared similar between nests that contained ants and those without (the variable was dropped from models with ΔAICc < 2; Tables 3 and 5).

The length of the primary feathers was greater in older nestlings from larger broods that were raised later in the spring (Fig. 2b–d; Tables 3, 4). The body mass of nestlings showed a strong increase only with their age and was greatest in 2019 (Fig. 2e–f; Tables 5, 6). Nestling growth was similar in deciduous and mixed deciduous-coniferous stands, as habitat type was excluded from the models with ΔAICc < 2 (Table 3) or became insignificant in averaged models (Tables 5–6).

### Mortality of Wood Warbler nestlings

Wood Warbler brood reduction (i.e. nestling mortality) from hatching to fledging was uncommon, occurring in 11 of 86 successful nests in total (two of 35 successful nests in 2018, four of 16 nests in 2019, and five of 35 in 2020). The difference between the number of nestlings fledged and hatched was comparable between nests with blowflies (mean 0.15, 95% CIs: 0.04–0.30, *n* = 27 nests) and those without (mean 0.22, 95% CIs: 0.06–0.43, *n* = 59).

## Discussion

The results showed an inconsistent relationship between weather conditions during the two stages of Wood Warbler nestling development and blowfly infestation within the dome-shaped nests of the birds. In the early nestling stage, when chicks were young and adult female blowflies usually lay their eggs in bird nests^14,25,27^, the likelihood of blowfly infestation increased if high ambient temperatures and low precipitation prevailed. Almost all of the nests infested with blowflies were exposed to ambient temperatures exceeding 15 °C, with little rain in the early nestling stage (Fig. 1a,b). A similar relationship was weak when weather conditions in the late nestling stage were tested. The intensity of blowfly infestation was unrelated to weather in the early nestling stage. However, in the late nestling stage, the number of blowflies was greater in infested nests that had been exposed to higher ambient temperatures during this period, but only if ants were absent from the same nests.

Moreover, the relationship between blowfly infestation and relative hatching date of Wood Warblers, or study year, appeared important only when the mean ambient temperature was dropped from the top hurdle models (on count or binomial parts). Thus, the increasing occurrence and intensity of blowfly infestation over a spring-summer breeding season, and the lowest occurrence of ectoparasites in 2020, seemed to also be related to ambient temperatures, which varied in the same manner within and between years (Supplementary Fig. S1, Supplementary Table S1). In contrast to some previous studies^22,24,29,33^, blowfly infestation changed little in relation to Wood Warbler brood size or habitat type (deciduous vs mixed deciduous-coniferous stands). Furthermore, despite some variation in nest infestation of ectoparasites, Wood Warbler nestling growth was unrelated to blowfly abundance, although it tended to be generally lower at high rainfall.

Our observations supported the hypothesis that warm and dry weather promotes ectoparasite infestation of bird nests. The results were consistent with the study by Merino and Potti^22^, who recorded the lowest blowfly parasitism of Pied Flycatcher *Ficedula hypoleuca* nests during the coldest and wettest breeding season. Eeva and Klemola^46^ also reported lower prevalence of *Protocalliphora* spp. in Pied Flycatcher nests if weather was cooler in the nestling period. A positive relationship between the intensity of blowfly infestation and ambient temperature was found in Blue Tit *Cyanistes caeruleus* nests in Corsica^23^ and in Spain^24^. Other studies showed inconsistent or insignificant relationships between weather during the nestling stage and blowfly nest infestation^33,47^, but much wider time-windows were used, so probably a poorer match with the timing of greatest sensitivity of blowflies.

The more frequent blowfly infestation of Wood Warbler nests that had been exposed to warm and dry weather in the early nestling stage could be related to increased activity of adult female blowflies at ambient temperatures exceeding 15 °C, probably when seeking dry nests in which to oviposit^26,27,30^. Warm weather might also influence the behaviour of a parent bird to shorten the brooding sessions and extend the time spent outside the nest^48^. The changes in brooding activity of the birds may create more opportunities for adult blowfly females to oviposit on small, unfeathered nestlings, left alone by the parent. However, the elevated intensity of blowfly infestation in nests that had been exposed to warm weather in the late nestling stage, when larvae develop in bird nests, might be due to increased survival of larvae in warmer nests^29^. Higher precipitation seemed to have no apparent impact on ectoparasite numbers, as the intensity of blowfly infestation remained stable in Wood Warbler nests regardless of rainfall levels, similar to an experimental study with dry and wetted nests^30^.

Although nests are composed of insulating materials and are warmed from within by the host birds, the nest temperature strongly depends on ambient temperature^21,49,50^. Thus, ambient conditions still could have a direct impact on developing blowfly larvae within unenclosed dome-shaped nests, with broadly similar insulative protection as unenclosed, open-cup nests^15–17^. As such, weather is likely to be an important proximate factor driving the prevalence and intensity of ectoparasite infestation in a range of bird nests.

As blowfly numbers were lower in nests containing ants, compared to nests without ants, the presence of the predatory insects might improve nest sanitation, particularly in warm weather when foraging ant workers could be more active^37,38^. Although ant predation on blowflies has not been directly observed in this study, predatory ants removing other ectoparasites from bird nests has been previously documented^36^. As such, a similar situation might have occurred in our study system, as predatory ant species were present in the warbler nests. Alternatively, the lower number of ectoparasites in nests with ants might result from reasons other than ant predation, such as chemical defence by ants towards different invertebrate species^51^. However, the developing blowfly larvae, as obligatory blood-sucking ectoparasites of birds, would be unlikely to escape from bird nests in this situation.

While more studies are needed to confirm the importance of ants in the sanitation of bird nests from ectoparasites, such intricate and weather-dependent mutualistic interactions might well occur between nesting birds and ants^21,52,53^. A previous experimental study indicated that the body heat of birds can attract ants to colonise bird nests to raise their own broods (larvae or pupae) within the nest structure^21^. Ant colonisation of bird nests could be driven by the benefits of raising broods under more suitable thermal conditions than in the ants’ own nests, particularly in cool and rainy weather^53^. Thus, active nests of endothermic birds could be an important source of warm nesting-sites that are sought by ants in cool regions^21,38^. Nevertheless, the current study suggests that the greatest potential benefits for birds hosting ants in their nests might occur in warm weather, when foraging ant workers may be active and potentially reduce the number of blowfly ectoparasites most effectively^36,37^. If correct, weather might have contradictory impacts on the interspecific interactions between the nest owners (birds) and commensal ants, with shifting benefits for both taxa under varying ambient conditions. Further work would be worthwhile to confirm the plausible weather-dependent symbiotic interactions between nesting birds and ants, which might be another example of ecological links that are potentially sensitive to climate change^38^.

Although cool weather and ant presence was associated with lower numbers of blowflies within infested warbler nests, contrary to our expectations there was no obvious relationship between the degree of blowfly infestation or ant presence and the growth or mortality of Wood Warbler nestlings. An insignificant impact of ectoparasites on songbird nestlings has also been reported in previous studies^24,25,46,54^, suggesting that impacts were undetected or that birds were able to compensate for the costs of parasitism^40,45,55,56^.

As blowfly larvae may aggregate and suck more blood from some of the nestlings in a brood, the detrimental impact of ectoparasites may be unevenly distributed among nestlings. This may consequently impede the detection of the relationship between blowfly abundance and the rate of nestling growth^57^. Alternatively, parent birds may be able to offset ectoparasite infestation, perhaps by frequent feeding rates when food is abundant, such as in the old-growth stands of the Białowieża Forest^58^. Nonetheless, blowfly infestation could have other negative effects on birds that we did not measure in this study. These effects could include anaemia^59,60^, increased begging intensity of nestlings that elevates predation risk^55^, or reduced survival or productivity of adults^33,61^. As such, blowfly parasitism may still be costly for host birds^14,25,31,55,60^.

The observed patterns of nestling growth in relation to rainfall could be due to direct effects of weather on birds by, for example, increasing heat loss and energy expenditure of nestlings in wet nests^39^, particularly in smaller broods^62^, or by interrupting food provisioning by parents^63^. However, high precipitation may also indirectly influence birds by reducing food availability, such as defoliating caterpillars or flying insects that constitute a large part of the diet of Wood Warbler nestlings^41–43,64,65^. As such, weather could affect the growth of nestlings in multiple ways. Although there is ample evidence of proximate weather impacts on the breeding success and survival of breeding birds^1^, understanding the influence of indirect impacts of ambient temperature or rainfall can be challenging, and requires further experimental approaches.

## Conclusions

In summary, our study provides novel information on the potential intricate impacts of weather on species that cohabit within the microenvironments of bird nests. Similar to other ecological networks^3,7,8^, ambient temperatures or rainfall appeared to directly influence the nest-dwelling arthropods and, to a lesser degree, the nesting birds. Additionally, weather conditions may also have cascading effects on host birds via interspecific interactions with ectoparasites and ants. However, further work is needed to confirm the role of ants in sanitising bird nests from ectoparasites at a range of ambient temperatures.

We found that the frequency of nest parasitism by blowflies was related to warm and dry weather in the early nestling stage, and blowflies were also more abundant in infested bird nests if higher ambient temperatures had occurred in the period of their larval development. As warm weather might improve nest sanitation by ants, the same ambient conditions may have conflicting impacts on ectoparasite abundance. However, neither the reduced nest infestation with blowflies, nor the presence of ants within bird nests, had a visible influence on warbler nestling growth or survival. The effect of weather on nestling growth was also rather weak, with only the length of primary feathers tending to be lower after high precipitation. Thus, birds seemed well able to tolerate unsuitable weather conditions and ectoparasite infestation in the old-growth stands of the Białowieża Forest, where superabundant food may help parents to compensate for the costs.

The study provides valuable information from a primeval temperate forest, little affected by direct human activity, which allows for future comparisons with investigations from human-altered habitats. The findings highlight the consequences of changing weather conditions for animals living within ecological networks, and may be particularly important in the era of climate change.

## Material and methods

### Study area

We conducted the study in the best-preserved stands of the Białowieża Forest, strictly protected within the Białowieża National Park (hereafter BNP; coordinates of Białowieża village: 52°42′N, 23°52′E). The extensive Białowieża Forest (*c*. 1500 km^2^) straddles the Polish-Belarusian border, where the climate is subcontinental with annual mean temperatures during May–July of 13–18 °C, and mean annual precipitation of 426–940 mm^66,67^.

The forest provides a unique opportunity to observe animals under conditions that likely prevailed across European lowlands before widespread deforestation and forest exploitation by humans^66,68,69^. The stands have retained a primeval character distinguished by a multi-layered structure, frequent fallen and standing dead trees, and a high species richness^66,70^. The stands are composed of about a dozen tree species of various ages, up to several hundred years old. The interspecific interactions and natural processes have been little affected by direct human activity.

We conducted observations mostly within the three permanent study plots (MS, N, W), totalling *c*. 130 ha, and in other nearby fragments of primeval oak-lime-hornbeam *Tilio-Carpinetum* or mixed deciduous-coniferous *Pino-Quercetum* stands. However, a small number of observations from adjacent managed deciduous forest stands were also included. For details of the study area see^71–73^.

### Study species

Our study system focused on ground-nesting Wood Warblers *Phylloscopus sibilatrix*, blowflies *Protocalliphora azurea,* and *Myrmica* or *Lasius* ants, which occurred in the birds’ nests.

The Wood Warbler is a small (*c*. 10 g) insectivorous songbird that winters in equatorial Africa and breeds in temperate European forests, typically rearing one or two broods each year^74^. Wood Warblers build dome-shaped nests for each breeding attempt, composed of woven grass, leaves and moss, and lined with animal hair^73^. The nests are situated on the ground among moderately sparse vegetation, often under a tussock of vegetation or near a fallen tree-branch or log (see examples in Supplementary Fig. S2)^53,75^. The breeding season of Wood Warblers begins in late April–early May and ends in July–August, when nestlings from replacement clutches (after initial loss) or second broods leave the nest. The typical clutch size in BNP is 5–7 eggs, and the nestling stage lasts 12–13 days^74,76^.

Wood Warbler nests are inhabited by various arthropods, including *Myrmica ruginodis* or *M. rubra* ants*,* and less often *Lasius platythorax, L. niger* or *L. brunneus*. The ants foraged and/or raised their own broods within the Wood Warbler nests^52^. The *Myrmica* and *Lasius* ant species are common in Europe^77,78^. Their colonies contain from tens to thousands of workers, and can be found on the forest floor, e.g. in soil, within or under fallen dead wood, in patches of moss, or among fallen tree-leaves^53,77,78^. All of the ant species found in the Wood Warbler nests are predators of other arthropods^77,79,80^.

Blowflies, *Protocalliphora* spp., are obligatory blood-sucking (hematophagous) ectoparasites that reproduce within bird nests. The occurrence, abundance, and impact of blowflies on Wood Warbler offspring is largely unknown, similar to many other European songbirds that build dome-shaped nests. Adult blowflies emerge in late spring and summer to lay eggs on the birds’ nesting material or directly onto the skin of typically newly hatched nestlings^14,26^. The blowfly larvae hatch within two–three days, and develop in the structure of warm bird nests for another 6–15 days, during which they emerge intermittently to feed on host blood, before finally pupating within the nests^14,25–27^.

### Data collection

#### Nest monitoring and measurements of nestlings

We searched for Wood Warbler nests daily from late April until mid-July in 2018–2020, by following birds mainly during nest-building. Nests were assigned to a deciduous or mixed deciduous-coniferous habitat type, depending on the tree stand where they were found. We inspected nests systematically, according to the protocol described in Wesołowski and Maziarz^76^. The number of observer visits was kept to a minimum to reduce disruptions for birds or potential risks of nest predation.

We aimed to establish the dates of hatching (day 0 ± 1 day), nestlings vacating the nest (fledging; ± 1 day) or nest failure (± 1–2 days). When nestlings hatched asynchronously, the hatching date corresponded to the earliest record of nestling hatching. The dates of fledging or nest failure were the mid-dates between the last visit when the nestlings were present in the nest, and the following visit, when the nest was found empty. Nest failure was primarily due to predation, which is the main cause of the Wood Warbler nest losses in BNP^76,81^ and elsewhere in Europe^82,83^.

To assess fitness consequences for birds of variable weather conditions, blowfly abundance and/or ant presence, we measured nestling growth and determined brood reduction (i.e. the mortality of chicks in the nest) from hatching until fledging. To define brood reduction, we assessed the number of hatchlings (nestlings up to 4 days old) and the number of fledglings leaving the nests. To ensure accurate counting and avoid premature fledging of nestlings, we established the number of fledglings on the day of measurement, when all nestlings were temporarily extracted from the nest.

We measured nestling growth on a single occasion when they were 6–9 days old (median 8 days), almost fully developed but too young to leave the nest. The measurements lasted for less than 10–15 min at each nest to minimise any potential risk of attracting predators. For each nestling we measured (using a ruler) the emerged length of the longest (3rd) primary feather vane (± 0.5 mm) on the left wing^84,85^, and body mass to the nearest 0.1 g using an electronic balance. The length of the feather vane is closely linked to feather growth^86^ and is one of the characteristics of nestling growth^85,87^. We treated the length of the primary feather vane and body mass as indices of nestling growth rate under varying conditions of weather, blood-sucking ectoparasites, or ant presence.

#### Extraction of arthropods from bird nests

To assess the number of blowflies and to establish the presence of ants, we checked the contents of 129 nests (including 11 nests from the managed forest stands) at which Wood Warbler nestlings had been measured. The sample included 86 successful breeding attempts (where a minimum of one nestling successfully left the nest), 27 failed (predated) nests (remnants of nestlings were found, but the nest structure remained intact), and 16 nests with an unknown fate (nestlings were large, so were capable of leaving the nest, but no family were located or other signs indicating fledging).

Due to ethical reasons, we were unable to collect the Wood Warbler nests and extract the ectoparasites and ants from them while they were in use by the birds. Removing the nests and replacing them with dummy nests would cause unacceptable nest desertion by adults. Therefore, we assessed the occurrence and number of blowflies or ant presence after Wood Warbler nestlings fledged or the breeding attempts failed naturally. We retrospectively explored the changes in blowfly infestation^14^, including the effect of ant presence^53^ in the same nests.

We collected nests from the field as soon as a breeding attempt ended, within approximately five days (median 1 day) following fledging or nest failure (nest structure remained intact). The delay of nest collection would not bias the ectoparasite infestation, as blowfly larvae pupate within bird nests and stay there after the hosts abandon their nests; puparia can be still found in nests collected in autumn or winter^14^. As the likelihood of finding ant broods (larvae or pupae associated with workers) was rather stable with the delay of nest collection^53^, the method seemed reliable also for assessing the presence of ant broods (35 of all 71 Wood Warbler nests containing ants). Only the number of nests with lone foraging ant workers could be underestimated, potentially inflating the uncertainty of tested relationships. However, as ants usually re-use rich food resources^88^, foraging *Myrmica* or *Lasius* ant workers might regularly exploit warbler nests, increasing the chances of finding the insects in the collected nests.

Wood Warbler nests were collected in one piece, with each placed into a separate sealed and labelled plastic bag. We carefully inspected the leaf litter around the nests, and the soil surface under them, to make sure that all blowfly larvae or pupae were collected. We transported the collected nests to a laboratory, where we stored them in a fridge for up to 5–6 days before the arthropod extraction.

To establish the number of blowflies and the presence of ants, in 2018, we carefully pulled apart the nesting material and searched for the arthropods amongst it ^52^. We gathered all blowfly pupae or larvae and a sample of ant specimens into separate tubes, labelled and filled with 70–80% alcohol, for later species identification. For nests collected in 2019–2020, we extracted the arthropods with a Berlese-Tullgren funnel. During the extraction, which usually lasted for 72 h, each nest was covered with fine metal mesh and placed *c*. 15 cm under the heat of a 40 W electric lamp. The arthropods were caught in 100 ml plastic bottles containing 30 ml of 70–80% ethanol, installed under each funnel. After the arthropod extraction, we carefully inspected the nesting material in the same way as in 2018, to collect any blowflies that remained within the nests. The quality of information collected on the number of ectoparasites and ant presence should be comparable each year.

#### Weather data

We obtained the mean daily temperatures and rainfall sums from a meteorological station, operated by the Meteorology and Water Management National Research Institute in the Białowieża village, 1–7 km from the study areas.

### Data analyses

#### Weather conditions affecting blowfly ectoparasites

To explore the impact of weather on blowfly ectoparasites, for each Wood Warbler nest we calculated average temperatures from daily means, and total sums of rainfall from daily sums, for the two time-windows in which we assumed the impact of weather would be of greatest importance:

i. the early nestling stage, when Wood Warbler nestlings were 1–4 days old. During this stage, female blowflies require a minimum temperature of *c*. 16 °C to become active and oviposit in bird nests^27^. Thus, cool and wet weather in the early nestling stage should reduce the activity of ovipositing blowflies, leading to less frequent ectoparasite infestation of Wood Warbler nests.
ii. The late nestling stage, when the warbler nestlings were aged between over four days old and until fledging or nest failure. During this stage, blowfly larvae grow and develop in bird nests after hatching a few days after oviposition^14,25–27^. As the temperature of bird nests strongly depends on ambient temperatures^21^, mortality of blowfly larvae should increase in cool weather, resulting in fewer ectoparasites in nests collected shortly after the fledging of birds^29^.

#### Weather conditions affecting Wood Warbler nestling growth

To explore the impact of weather on nestling growth, for each nest we calculated the average temperatures and total sums of rainfall for the period when nestlings were over four days old and until their measurement, usually on day 8 from hatching (see above). During this stage, nestlings are no longer brooded by a parent^74^, so must balance their energetic expenditure between growth (feather length and body mass) or thermoregulation^89^. Thus, we expected that the gain in body mass and the growth of flight feathers would be reduced in nestlings during cool and wet weather, when maintaining a stable body temperature would be costly^90^.

### Statistical analyses

All statistical tests were two-tailed and performed in R version 4.1.0^91^.

#### The changes in blowfly infestation of the Wood Warbler nests

To test the changes in blowfly infestation of warbler nests, we used zero-augmented negative binomial models (package pscl in R;^92,93^), which deal with the problem of overdispersion and excess of zeros^92^. In this study, hurdle and zero-inflated models fitted with the same covariates had an almost identical Akaike Information Criterion (AIC). Therefore, we presented only the results of hurdle models, which are easier to interpret than zero-inflated models. Hurdle models consisted of two parts: a left-truncated count with a negative binomial distribution representing the number of blowflies in infested nests, and a zero hurdle binomial estimating the probability of blowfly presence. We used models with a negative binomial distribution, which had a much lower AIC than with a Poisson distribution on a count part.

We designed the most complex (global) model that contained a response variable of the number of blowflies in each of the 129 Wood Warbler nests. The covariates were: mean ambient temperature, total sum of rainfall, presence (or absence) of ants in the same nests, habitat type (deciduous vs mixed deciduous-coniferous forest), study year (2018–2020), the number of nestlings hatched (brood size), and nest phenology (the relative hatching date of Wood Warbler nestlings, as days from the median hatching date in a season: 23 May in 2018, 25 May in 2019 and 29 May in 2020). The initial global model also contained the two-way interaction terms that we suspected to be important: between temperature and rainfall, temperature and presence of ants, and rainfall and presence of ants.

To explore all potentially meaningful subsets of models, we used the same covariates on both parts (count and binomial) of the global model. We performed automated model selection with the MuMIn package^94^, starting from the most complex (global) model and using all possible simpler models (i.e. all subsets)^95^. To attain the minimum sample size of *c*. 20 data points for each parameter^96^, we limited the maximum number of parameters to six in each part (count or binomial) of the candidate models.

As some of the interaction terms appeared insignificant in the initial model selection, to minimise the risk of over-parametrisation, we included only the significant interaction term on a count part of the final global model. As described above, we performed model selection again. We tested linear relationships, as the quadratic effects of weather variables (presuming temperature or rainfall optima) appeared insignificant.

To test whether blowfly infestation changed with weather in the early or late nestling stages, we twice repeated the procedure described above. The first global model included the mean ambient temperature and the total sum of rainfall for the early nestling stage, and the second global model contained weather variables for the late nestling stage. The remaining covariates were the same.

A practice of including the same sets of covariates on count and binomial parts has been previously questioned^97^. However, our approach allowed us to comply with these objections^97^, as we presented only the most parsimonious models (with ΔAICc < 2) which all contained different sets of covariates on count and binomial parts.

We considered models differing by less than two AICc units from the best fitting model (with smallest AICc) as equally informative^98^. We averaged the top models, always with different sets of covariates on count and binomial parts, by using conditional averaging, to obtain parameter estimates and their 95% CIs reflecting model selection uncertainty.

The analyses comprised successful (*n* = 86) and other nests that failed in the late nestling stage (nest structure remained intact; *n* = 27) or their fate was unknown (nestlings were large enough to escape a predator attack; *n* = 16). Including failed nests or those with the unknown status increased sample sizes, and was justified because the same patterns emerged when only the successful breeding attempts were analysed.

We calculated bootstrapped means and 95% CIs (10,000 random re-samplings), to present the number of blowflies in infested nests with and without ants, or in different years.

#### The growth of Wood Warbler nestlings

To explore the changes in the growth of Wood Warbler nestlings with varying weather conditions, blowfly abundance, ant presence, or other factors (relative hatching date, brood size, nestling age, habitat type, study year), we used linear mixed effect models, with package glmmTMB^99^. In the analysis we employed the data from 2018 to 2020 for the length of the third primary feather and body mass of 742 nestlings from 129 Wood Warbler nests. The models contained a random intercept for nest identity that accounted for non-independent growth rates of nestlings from the same nests.

We modelled the length of the third primary feather or body mass, both assessed *c*. 8 days post-hatching, as responses to nine predictors: mean ambient temperature and total sum of rainfall in the stage when nestlings were over four days old and until their measurement, blowfly abundance (i.e., the number of blowflies per nestling in nests with and without blowflies), presence or absence of ant workers, and also habitat type (deciduous vs mixed deciduous-coniferous), study year, relative hatching date of birds, brood size, and nestling age (number of days since hatching on day 0). In models, we included nestling age to correct for deviations from the intended measurement age at 8 days post-hatching.

For both measures of nestling growth (feather growth and body mass), global models initially contained main effects of all covariates mentioned above and the potentially important two-way interaction terms between temperature and rainfall, temperature and blowfly abundance, rainfall and blowfly abundance, and temperature and ant presence. However, as none of the interaction terms appeared significant, we tested only the main effects. All models included only linear relationships, as none of the quadratic effects (presuming temperature or rainfall optima) were significant.

To create a subset of models with variables best explaining the growth of Wood Warbler nestlings, we used an automated model selection in MuMIn^94^, according to an information-theoretic approach. This method allowed us to find the most parsimonious models among the candidate model set, which were drawn from the most complex (global) model^98^. To attain the minimum sample size of *c*. 20 data points for each estimated parameter^96^, we limited the maximum number of parameters to six in the candidate models. We considered models differing by less than two AICc units from the best fitting model (with smallest AICc) as equally informative^98^. We averaged these top models (using conditional averaging) to obtain parameter estimates and their 95% CIs reflecting model selection uncertainty.

#### Mortality of Wood Warbler nestlings

To test whether the nest infestation with blowflies increased the mortality of Wood Warbler nestlings, we compared brood reduction (i.e. the difference between the number of fledglings and hatchlings) in 86 successful nests with and without ectoparasites, based on bootstrapped means and 95% CIs (10,000 random resamplings).

## Supplementary Information

Supplementary Information.

## Data Availability

The datasets analysed during the current study are available in the Figshare repository, 10.6084/m9.figshare.21229472.v1^100^.

## References

[1] Newton I (1998). Population Limitation in Birds.

[2] Ottersen G (2001). Ecological effects of the North Atlantic Oscillation. Oecologia.

[3] Hoover SER, Tylianakis JM, Candolin U, Wong BBM (2012). Species interactions. Behavioural Responses to a Changing World: Mechanisms and Consequences.

[4] Parmesan C, Root TL, Willig MR (2000). Impacts of extreme weather and climate on terrestrial biota. Bull. Am. Meteor. Soc..

[5] Maxwell SL (2019). Conservation implications of ecological responses to extreme weather and climate events. Divers. Distrib..

[6] Pocock MJO, Evans DM, Memmott J (2012). The robustness and restoration of a network of ecological networks. Science.

[7] Mouritsen KN, Poulin R (2002). Parasitism, climate oscillations and the structure of natural communities. Oikos.

[8] Czeszczewik D (2020). Climate change has cascading effects on tree masting and the breeding performance of a forest songbird in a primeval forest. Sci. Total Environ..

[9] Woodroffe GE (1953). An ecological study of the insects and mites in the nests of certain birds in Britain. Bull. Entomol. Res..

[10] Krištofík J, Šustek Z, Gajdoš P (1995). Arthropods in the penduline tit (*Remiz pendulinus*) nests: occurrence and abundance in different breeding phases. Biol. Bratisl..

[11] Dobroscky ID (1925). External parasites of birds and the fauna of birds' nests. Biol. Bull. Mar. Biol. Lab..

[12] Deeming DC, Reynolds SJ (2015). Nests, Eggs, and Incubation. New Ideas About Avian Reproduction.

[13] Collias NE, Collias EC (1984). Nest Building and Bird Behavior.

[14] Saborsky CW, Bennett GF, Whitworth TL (1989). Bird Blow Flies (Protocalliphora) in North America (Diptera: Calliphoridae), with Notes on the Palearctic Species.

[15] Skowron C, Kern M (1980). The insulation in nests of selected North American songbirds. Auk.

[16] Deeming DC, Biddle LE (2015). Thermal properties of bird nests depend on air-gaps between the materials. Acta Ornithol..

[17] Griffith SC, Mainwaring MC, Sorato E, Beckmann C (2016). High atmospheric temperatures and ‘ambient incubation’ drive embryonic development and lead to earlier hatching in a passerine bird. R. Soc. open sci..

[18] Martin TE (2017). Enclosed nests may provide greater thermal than nest predation benefits compared with open nests across latitudes. Funct. Ecol..

[19] Matysioková B, Remeš V (2018). Evolution of parental activity at the nest is shaped by the risk of nest predation and ambient temperature across bird species. Evolution.

[20] Perez DM, Gardner JL, Medina I (2020). Climate as an evolutionary driver of nest morphology in birds: a review. Front. Ecol. Evol..

[21] Maziarz M (2020). Thermal ecosystem engineering by songbirds promotes a symbiotic relationship with ants. Sci. Rep..

[22] Merino S, Potti J (1996). Weather dependent effects of nest ectoparasites on their bird hosts. Ecography.

[23] Mennerat, A., Charmantier, A., Perret, P., Hurtrez-Boussès, S. & Lambrechts, M. M. Parasite intensity is driven by temperature in a wild bird. *bioRxiv 323311, ver. 4 peer-reviewed and recommended by PCI Ecology*, 10.1101/323311 (2019).

[24] Castaño-Vázquez F, Merino S (2021). Differential effects of environmental climatic variables on parasite abundances in blue tit nests during a decade. Integr. Zool..

[25] Whitworth, T. L. *Host and Habitat Preferences, Life History, Pathogenicity and Population Regulation in Species of Protocalliphora Hough (Diptera: Calliphoridae). All Graduate Theses and Dissertations. 5059*, (1976).

[26] Gold CS, Dahlsten DL (1989). Prevalence, habitat selection, and biology of *Protocalliphora* (Diptera: Calliphoridae) found in nests of mountain and chestnut-backed chickadees in California. Hilgardia.

[27] Bennett GF, Whitworth TL (1991). Studies on the life history of some species of *Protocalliphora* (Diptera: Calliphoridae). Can. J. Zool..

[28] Maziarz M (2019). Breeding birds actively modify the initial microclimate of occupied tree cavities. Int. J. Biometeorol..

[29] Dawson RD, Hillen KK, Whitworth TL (2005). Effects of experimental variation in temperature on larval densities of parasitic *Protocalliphora* (Diptera: Calliphoridae) in nests of tree swallows (Passeriformes: Hirundinidae). Environ. Entomol..

[30] Heeb P, Kölliker M, Richner H (2000). Bird-ectoparasite interactions, nest humidity, and ectoparasite community structure. Ecology.

[31] Owen DF (1954). *Protocalliphora* in birds' nests. Br. Birds.

[32] Hurtrez-Boussès S, de Garine-Wichatitsky M, Perret P, Blondel J, Renaud F (1999). Variations in prevalence and intensity of blow fly infestations in an insular Mediterranean population of blue tits. Can. J. Zool..

[33] Wesołowski T (2001). Host-parasite interactions in natural holes: marsh tits (*Parus palustris*) and blow flies (*Protocalliphora falcozi*). J. Zool Lond..

[34] Bush SE, Clayton DH (2018). Anti-parasite behaviour of birds. Philos. Trans. R. Soc. Lond. B Biol. Sci..

[35] Brown CR, Page CE, Robison GA, O’Brien VA, Booth W (2015). Predation by ants controls swallow bug (Hemiptera: Cimicidae: *Oeciacus vicarius*) infestations. J. Vector Ecol..

[36] Salido A, Veiga J, Reyes-López JL, Nieves-Aldrey JL, Valera F (2021). Insect predation reduces the abundance of a nidicolous ectoparasite. Ecol. Entomol..

[37] Hölldobler B, Wilson EO (1990). The Ants.

[38] Parr CL, Bishop TR (2022). The response of ants to climate change. Glob. Chang Biol..

[39] Mertens JAL (1977). Thermal Conditions for successful breeding in Great Tits (*Parus major* L.) II. Thermal properties of nests and nestboxes and their implications for the range of temperature tolerance of great tit broods. Oecologia.

[40] Dufva R, Allander K (1996). Variable effects of the Hen Flea *Ceratophyllus gallinae* on the breeding success of the Great Tit *Parus major* in relation to weather conditions. Ibis.

[41] Cox AR, Robertson RJ, Lendvai AZ, Everitt K, Bonier F (2019). Rainy springs linked to poor nestling growth in a declining avian aerial insectivore (*Tachycineta bicolor*). Proc. Biol. Sci..

[42] Bryant DM (1975). Breeding biology of House Martins *Delichon urbica* in relation to aerial instect abundance. Ibis.

[43] Keller LF, van Noordwijk AJ (1994). Effects of local environmental conditions on nestling growth in the Great Tit *Parus major* L.. Ardea.

[44] Simon A, Thomas D, Blondel J, Perret P, Lambrechts MM (2004). Physiological ecology of Mediterranean blue tits (*Parus caeruleus* L.): effects of ectoparasites (*Protocalliphora* spp.) and food abundance on metabolic capacity of nestlings. Physiol. Biochem. Zool..

[45] Senécal S (2021). Poor prey quality is compensated by higher provisioning effort in passerine birds. Sci. Rep..

[46] Eeva T, Klemola T (2013). Variation in prevalence and intensity of two avian ectoparasites in a polluted area. Parasitology.

[47] Musgrave K, Bartlow AW, Fair JM (2019). Long-term variation in environmental conditions influences host-parasite fitness. Ecol. Evol..

[48] Johnson EJ, Best LB (1982). Factors affecting feeding and brooding of gray catbird nestlings. Auk.

[49] Ricklefs RE, Hainsworth FR (1969). Temperature regulation in nestling Cactus Wrens: the nest environment. Condor.

[50] Deeming DC, Pike TW (2015). Nest surface temperature predicts fledging success of Blue Tits *Cyanistes caeruleus* but not Great Tits *Parus major*. Acta Ornithologica.

[51] Bell WJ, Cardé RT (1984). Chemical Ecology of Instects.

[52] Maziarz M, Broughton RK, Hebda G, Wesołowski T (2018). Occupation of wood warbler *Phylloscopus sibilatrix* nests by *Myrmica* and *Lasius* ants. Insectes Soc..

[53] Maziarz M (2021). Interspecific attraction between ground-nesting songbirds and ants: the role of nest-site selection. Front. Zool..

[54] Johnson LS, Albrecht DJ (1993). Effects of haematophagous parasites on nestling house wrens, *Troglodytes aedon*: Who pays the cost of parasitism?. Oikos.

[55] Christe P, Richner H, Oppliger A (1996). Begging, food provisioning, and nestling competition in great tit broods infested with ectoparasites. Behav. Ecol..

[56] Bańbura J (2004). Effects of *Protocalliphora* parasites on nestling food composition in Corsican Blue Tits *Parus caeruleus*: consequences for nestling performance. Acta Ornithologica.

[57] Simon A, Thomas DW, Blondel J, Lambrechts MM, Perret P (2003). Within-brood distribution of ectoparasite attacks on nestling blue tits: a test of the tasty chick hypothesis using inulin as a tracer. Oikos.

[58] Wesołowski T (2007). Primeval conditions - what can we learn from them?. Ibis.

[59] Whitworth TL, Bennett GF (1992). Pathogenicity of larval *Protocalliphor*a (Diptera: Calliphoridae) parasitizing nestling birds. Can. J. Zool..

[60] Hurtrez-Boussès S, Blondel J, Perret P, Fabreguettes J, Renaud F (1998). Chick parasitism by blowflies affects feeding rates in a Mediterranean population of blue tits. Ecol. Lett..

[61] Møller AP, Allander K, Dufva R, Blondel J, Gosler A, Lebreton J-D, McCleery R (1990). Fitness effects of parasites on passerine birds: a review. Population Biology of Passerine Birds.

[62] Royama T (1966). Factors governing feeding rate, food requirement and brood size of nestling Great Tits *Parus major*. Ibis.

[63] Arlettaz R, Schaad M, Reichlin TS, Schaub M (2010). Impact of weather and climate variation on Hoopoe reproductive ecology and population growth. J. Ornithol..

[64] Maziarz M, Wesołowski T (2010). Timing of breeding and nestling diet of Wood Warbler *Phylloscopus sibilatrix* in relation to changing food supply. Bird Study.

[65] Mallord JW (2017). Diet flexibility in a declining long-distance migrant may allow it to escape the consequences of phenological mismatch with its caterpillar food supply. Ibis.

[66] Faliński JB (1986). Vegetation Dynamics in Temperate Lowland Primeval Forests. Ecological Studies in Białowieża Forest.

[67] Boczoń A, Kowalska A, Ksepko M, Sokołowski K (2018). Climate warming and drought in the Bialowieza forest from 1950–2015 and their impact on the dieback of Norway spruce stands. Water.

[68] Tomiałojć L, Wesołowski T, Walankiewicz W (1984). Breeding bird community of a primaeval temperate forest (Białowieża National Park, Poland). Acta Ornithologica.

[69] Jaroszewicz B (2019). Białowieża Forest—A relic of the high naturalness of European forests. Forests.

[70] Bobiec A (2002). Living stands and dead wood in the Białowieża forest: suggestions for restoration management. For. Ecol. Manage..

[71] Wesołowski T (2015). 40 years of breeding bird community dynamics in a primeval temperate forest (Białowieża National Park, Poland). Acta Ornithologica.

[72] Broughton RK, Bubnicki JW, Maziarz M (2020). Multi-scale settlement patterns of a migratory songbird in a European primaeval forest. Behav. Ecol. Sociobiol..

[73] Napierała A (2021). Lack of specialist nidicoles as a characteristic of mite assemblages inhabiting nests of the ground-nesting wood warbler, *Phylloscopus sibilatrix* (Aves: Passeriformes). Exp. Appl. Acarol..

[74] Cramp S (1992). The Birds of the Western Palearctic.

[75] Wesołowski T (1985). The breeding ecology of the Wood Warbler *Phylloscopus sibilatrix* in primaeval forest. Ornis Scand..

[76] Wesołowski T, Maziarz M (2009). Changes in breeding phenology and performance of Wood Warblers *Phylloscopus sibilatrix* in a primeval forest: a thirty-year perspective. Acta Ornithologica.

[77] Radchenko A, Czechowski W, Czechowska W (1997). The genus *Myrmica* Latr. (Hymenoptera, Formicidae) in Poland – a survey of species and a key for their identification. Ann. Zool..

[78] Czechowski W, Radchenko A, Czechowska W (2002). The Ants (Hymenoptera, Formicidae) of Poland.

[79] Šipoš J, Drozdová M, Drozd P (2013). Assessment of trends in predation pressure on insects across temperate forest microhabitats. Agric. For. Entomol..

[80] Bulgarini G, Castracani C, Mori A, Grasso DA, Maistrello L (2021). Searching for new predators of the invasive *Halyomorpha halys*: the role of the black garden ant *Lasius niger*. Entomol. Exp. Appl..

[81] Maziarz M (2019). Patterns of predator behaviour and Wood Warbler *Phylloscopus sibilatrix* nest survival in a primaeval forest. Ibis.

[82] Grendelmeier A, Arlettaz R, Gerber M, Pasinelli G (2015). Reproductive performance of a declining forest passerine in relation to environmental and social factors: implications for species conservation. PLoS ONE.

[83] Bellamy PE (2018). Nest predation and the influence of habitat structure on nest predation of Wood Warbler *Phylloscopus sibilatrix*, a ground-nesting forest passerine. J. Ornithol..

[84] Svensson L (1992). Identification Guide to European Passerines.

[85] Kania W (1983). Probability method of ageing passerine nestlings and its usage in breeding phenology investigations of Starling. Notatki Ornitol..

[86] Zach R, Mayoh KR (1982). Weight and feather growth of nestling tree swallows. Can. J. Zool..

[87] Hałupka L (2018). Ageing nestlings of the Reed Warbler *Acrocephalus scirpaceus*. Ringing Migr..

[88] Csata E, Dussutour A (2019). Nutrient regulation in ants (Hymenoptera: Formicidae): a review. Myrmecol. News.

[89] Schew WA, Ricklefs RE, Starck JM, Ricklefs RE (1998). Developmental plasticity. Avian Growth and Development.

[90] Dawson RD, Lawrie CC, O'Brien EL (2005). The importance of microclimate variation in determining size, growth and survival of avian offspring: Experimental evidence from a cavity nesting passerine. Oecologia.

[91] R Core Team. R: A language and environment for statistical computing. R Foundation for Statistical Computing, Vienna, Austria. https://www.R-project.org/. (2021).

[92] Zeileis A, Kleiber C, Jackman S (2008). Regression models for count data in R. J. Stat. Softw..

[93] Jackman, S. pscl: Classes and methods for R developed in the political science computational laboratory. United States Studies Centre, University of Sydney. Sydney, New South Wales, Australia. R package version 1.5.5. URL https://github.com/atahk/pscl/. (2020).

[94] Barton, K. MuMIn: Multi-Model Inference. R package version 1.43.17. https://CRAN.R-project.org/package=MuMIn. (2020).

[95] Grueber CE, Nakagawa S, Laws RJ, Jamieson IG (2011). Multimodel inference in ecology and evolution: challenges and solutions. J. Evol. Biol..

[96] Harrell FE (2015). Regression Modeling Strategies. With Applications to Linear Models, Logistic and Ordinal Regression, and Survival Analysis.

[97] Kéry M, Royle JA (2016). Applied Hierarchical Modeling in Ecology: Analysis of Distribution Abundance and Species Richness in R and BUGS. Volume 1: Prelude and Static Models.

[98] Burnham KP, Anderson DR (2002). Model Selection and Multimodel Inference.

[99] Brooks ME (2017). glmmTMB balances speed and flexibility among packages for zero-inflated generalized linear mixed modeling. R J..

[100] Maziarz, M., Broughton, R. K., Chylarecki, P. & Hebda, G. Weather impacts on interactions between nesting birds, nest-dwelling ectoparasites and ants - dataset. figshare. Dataset. 10.6084/m9.figshare.21229472.v1 (2022). 10.1038/s41598-022-21618-1 PMC9596701 36284124

