# Supplementary material for: Weather impacts on interactions between nesting birds, nest-dwelling ectoparasites and ants
Source: Sci Rep. 2022 Oct 25;12:17845. doi: 10.1038/s41598-022-21618-1 (PMC9596701; doi:10.1038/s41598-022-21618-1)
Supplement: Supplementary file 1 — Supplementary Information. [file 41598_2022_21618_MOESM1_ESM.pdf]

Supplementary Material

Title: "Weather impacts on interactions between nesting birds, nest-dwelling ectoparasites and ants"

Authors: Marta Maziarz [a*], Richard K. Broughton [b], Przemysław Chylarecki [a], Grzegorz Hebda [c]

[a] *Museum and Institute of Zoology, Polish Academy of Sciences, Wilcza 64, 00-679 Warsaw, Poland, email: mmaziarz@miiz.waw.pl, ORCiD: 0000-0002-2921-5713; email: pch@miiz.waw.pl, ORCiD: 0000-0001-6863-4294*

[b] *UK Centre for Ecology & Hydrology, Maclean Building, Benson Lane, Crowmarsh Gifford, Wallingford, OX10 8BB, UK; email: rbrou@ceh.ac.uk, ORCiD: 0000-0002-6838-9628*

[c] *Institute of Biology, University of Opole, Oleska 22, 45 052 Opole, Poland; email: grzesio@uni.opole.pl, ORCiD: 0000-0002-8668-1809*

*Corresponding author

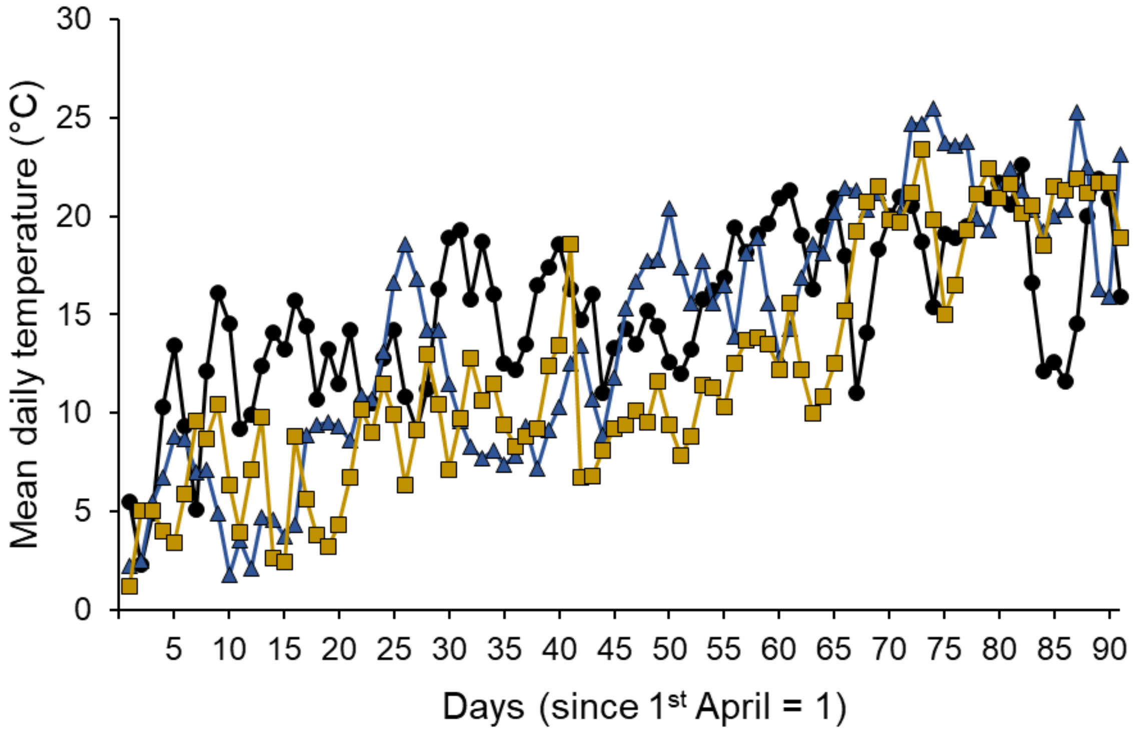

Figure S1. Mean daily ambient temperatures (°C) recorded by the meteorological station in the Białowieża village, between the 1st of April and the 30th of June in 2018 (●), 2019 (▲), or 2020 (■).

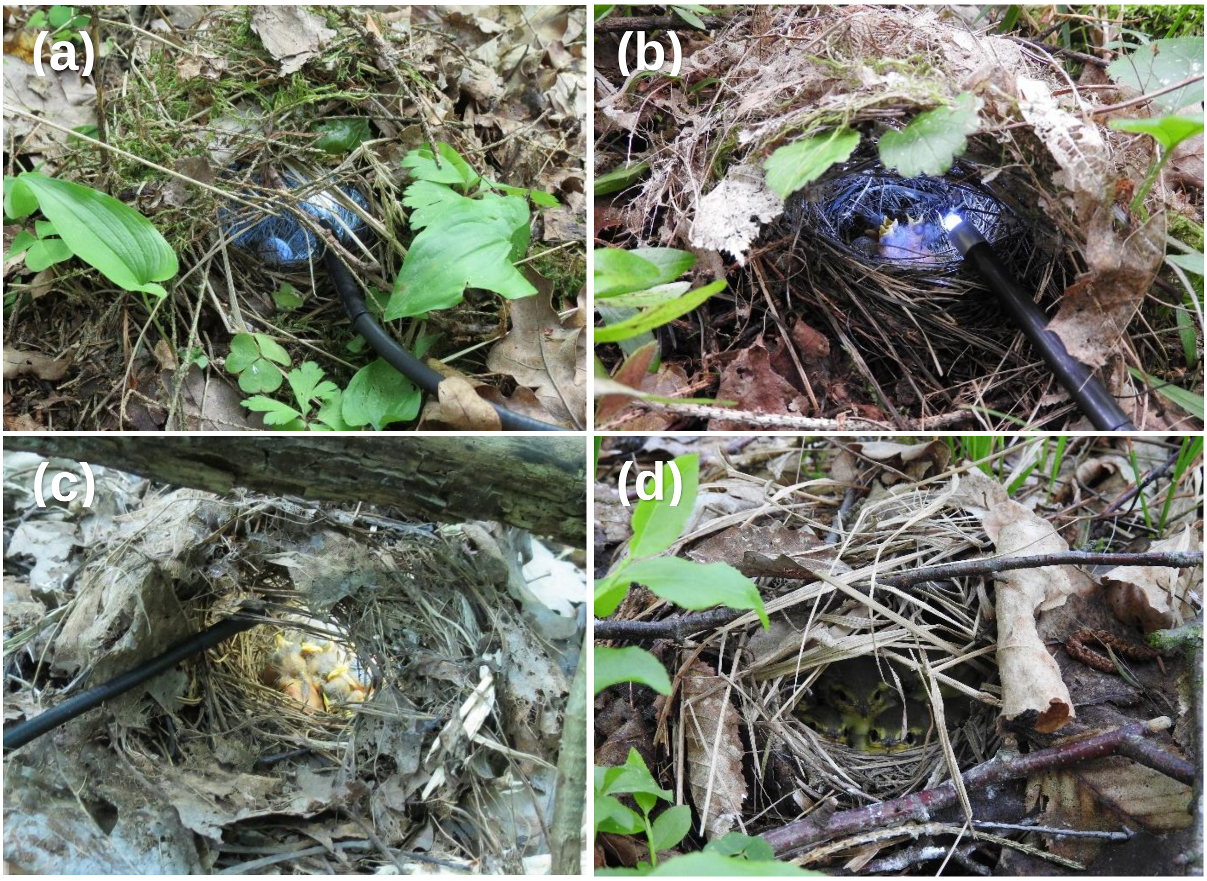

Figure S2. Examples of Wood Warbler *Phylloscopus sibilatrix* nests during: (**a**) the egg-laying stage, (**b**, **c**) early nestling stage (< 5 days post-hatching), and (**d**) late nestling stage (> 4 days post-hatching). Photos: Richard K. Broughton and Marta Maziarz.

Table S1. The annual changes in mean daily ambient temperatures (°C) and daily sums of rainfall (mm) in the period from the earliest hatching date of Wood Warbler nestlings to the latest fledging or nest failure date. Shown are bootstrapped means and 95% CIs (10,000 replications). Weather data came from the meteorological station in the Białowieża village.

Year	Temperature (°C)		Sum of rainfall (mm)		Date (1 = 1st April)	
	mean	95% CIs	mean	95% CIs	earliest hatching	latest fledging or nest failure
2018	17.4	16.3-18.4	0.6	0.1-1.2	47	77
2019	19.6	18.5-20.7	0.9	0.1-2.2	52	85
2020	15.7	14.2-17.2	3.4	1.4-6.0	48	83
